# Risk Factors for West Nile Virus Neuroinvasive Disease, California, 2005

**DOI:** 10.3201/eid1312.061265

**Published:** 2007-12

**Authors:** Cynthia M. Jean, Somayeh Honarmand, Janice K. Louie, Carol A. Glaser

**Affiliations:** * California Department of Public Health, Richmond, California, USA

**Keywords:** West Nile virus, California, neuroinvasive disease, risk factors, diabetes, dispatch

## Abstract

In 2005, 880 West Nile virus cases were reported in California; 305 case-patients exhibited neuroinvasive disease, including meningitis, encephalitis, or acute flaccid paralysis. Risk factors independently associated with developing neuroinvasive disease rather than West Nile fever included older age, male sex, hypertension, and diabetes mellitus.

Since the first identification of West Nile virus (WNV) in North America in New York, New York, in 1999, the virus has spread rapidly westward across the United States. In 2004 and 2005, California was the national epicenter of WNV activity, with 779 and 880 cases, respectively. The aim of this study was to identify potential risk factors for developing West Nile neuroinvasive disease among the WNV case-patients reported in California.

## The Study

WNV human surveillance in California is conducted through several different mechanisms. Local clinicians are asked to refer patients with evidence of WNV disease, including encephalitis, aseptic meningitis, acute flaccid paralysis, or illness compatible with West Nile fever, for testing which is performed by 33 local public health laboratories and the state Viral and Rickettsial Disease Laboratory (VRDL). Persons with suspected cases are also tested through the California Encephalitis Project (*1*), which provides enhanced diagnostic testing for several viral agents that cause encephalitis, including WNV. In addition, Kaiser Permanente laboratories screen patients with suspected cases and forward positive specimens to VRDL for further testing, while commercial reference laboratories forward positive test results. Blood collection centers forward reports of WNV-positive donors, and local health departments perform follow-up investigations to identify donors in whom clinical disease later develops.

Local health departments use a standardized case history form to collect demographic and clinical information about patients who meet the clinical and laboratory criteria for WNV infection. Patients are classified as having West Nile fever if they exhibit symptoms of WNV infection (e.g., fever, headache, or muscle weakness) without development of neurologic manifestations (e.g., encephalitis, meningitis, or acute flaccid paralysis). The case history form includes questions about hypertension and diabetes.

The 880 case-patients identified were reported from 40 of 58 counties in California, with illness onset ranging from May through November 2005. The median age of all case-patients was 50 years (range 2–95 years), compared to a median of 78 years for the 19 WNV patients who died (range 56–92 years; p<0.0001); 55% of all patients were male. Of the 880 cases, 534 cases were classified as West Nile fever and 305 as WNV neuroinvasive disease. Not surprisingly, a greater proportion of the patients with neuroinvasive disease were hospitalized (90%) and required intensive care (27%) compared with the West Nile fever patients (31% and 2%, respectively; p<0.0001). The neuroinvasive disease patients also reported a greater frequency of severe symptoms such as altered mental status (54%) and seizures (7%) than did the West Nile fever patients (15% and 0.7%; p<0.0001 and p<0.001, respectively). Rash was reported among 22% of neuroinvasive disease patients compared with 51% of West Nile fever patients (p<0.0001), possibly because those with more severe disease are less able to mount an inflammatory response (*2*) (Table 1).

**Table 1:** Characteristics of WNV cases reported in California, 2005*

Characteristic	WNND, no./total (%)	WNF, no./total (%)	95% Confidence interval	p value†
Sex, M	188/305 (62)	270/534 (51)	1.18–2.09	<0.01
Age, y				
<18	12/305 (4)	19/534 (4)	0.53–2.32	0.85
18–44	73/305 (24)	180/534 (34)	0.45–0.85	<0.01
45–64	121/305 (40)	240/534 (45)	0.61–1.07	0.15
65–74	51/305 (17)	61/534 (11)	1.09–2.33	0.03
>75	48/305 (16)	33/534 (6)	1.78–4.53	<0.0001
Symptom				
Fever	254/283 (90)	315/456 (69)	2.54–6.04	<0.0001
Headache	202/262 (77)	387/483 (80)	0.58–1.20	0.33
Rash	54/244 (22)	239/469 (51)	0.19–0.39	<0.0001
Muscle pain/weakness	215/274 (78)	393/485 (81)	0.59–1.23	0.40
Seizures	11/153 (7)	2/313 (0.6)	2.64–55.06	0.001
Altered mental status	148/275 (54)	68/463 (15)	4.77–9.61	<0.0001

*WNV, West Nile virus; WNND, West Nile neuroinvasive disease; WNF, West Nile fever. †Fisher exact test, 2-tailed.

A greater proportion of neuroinvasive disease patients (46%) reported hypertension as an underlying medical condition than did the West Nile fever patients (29%; p<0.001). Thirty-three percent of the neuroinvasive disease patients reported having diabetes mellitus, compared with 11% of the West Nile fever patients (p<0.001).

In response to an open-ended question about past medical history, 193 (22%) of all case-patients reported other underlying illnesses. Coronary vascular disease was the most common underlying condition in both neuroinvasive disease and West Nile fever patients (23% and 17%, respectively). Other medical conditions reported for patients with neuroinvasive disease included renal insufficiency (10%) and chronic obstructive pulmonary disorder (8%); cancer (10%) and asthma (9%) were more frequently reported for West Nile fever patients.

Using SAS version 9.1 software (SAS Institute, Inc., Cary, NC, USA), we conducted a univariate analysis to compare the characteristics of patients with neuroinvasive disease to those with West Nile fever (Table 2). Odds ratios (ORs) and 95% confidence intervals (CIs) were calculated by using Cochran-Mantel-Haenszel statistics. Patients with neuroinvasive disease were twice as likely to have hypertension (95% CI 1.44–3.01) and 4 times more likely to have diabetes (95% CI 2.63–6.55) than West Nile fever patients. Other risk factors for neuroinvasive disease included age >64 years (OR = 2.24, 95% CI 1.62–3.11) and male sex (OR = 1.57, 95% CI 1.18–2.09). Because of its collinearity with diabetes, hypertension was dropped from the logistic regression model. Age >64 years (p = 0.03), male sex (p<0.01), and diabetes (p<0.0001) were independently associated with neuroinvasive disease.

**Table 2:** Univariate analysis of potential risk factors for developing WNND versus WNF*

Characteristic	WNF, no. (%)	WNND, no. (%)	Odds ratio	95% Confidence interval
Diabetes	39 (11)	60 (34)	4.15	2.63–6.55
Age >64 y	94 (18)	99 (32)	2.24	1.62–3.11
Hypertension	105 (29)	83 (46)	2.08	1.44–3.01
Male sex	270 (51)	188 (62)	1.57	1.18–2.09

*WNND, West Nile neuroinvasive disease; WNF, West Nile fever.

## Conclusions

To our knowledge, this report summarizes the epidemiologic and clinical characteristics of the largest number of WNV case-patients to date. In contrast to most previous studies, our study included all patients identified with WNV illness, regardless of severity (i.e., inpatients and outpatients). Among the case-patients identified in our surveillance, univariate analysis identified older age and male sex as significant independent predictors of developing neuroinvasive disease, as seen in national surveillance data (*3*). Hypertension and diabetes were also identified as risk factors for developing neuroinvasive disease rather than West Nile fever. Notably, the frequency of diabetes is higher in patients with West Nile neuroinvasive disease than among the general population. The 2001 California Health Interview Survey, a population-based, standardized telephone health survey of >55,000 households throughout California, found that 1,225,000 (11%) persons in California >45 years of age reported ever having received a diagnosis of diabetes (*4*). In contrast, 44% of WNV neuroinvasive disease patients >45 years of age in our study had diabetes mellitus.

Others have cited diabetes and hypertension as possible risk factors for progression to West Nile neuroinvasive disease or death. In a study of 59 patients hospitalized with WNV infection in New York City (*5*), diabetes was an independent risk factor for death (age-adjusted relative risk = 5.1; 95% CI 1.5–17.3). A history of hypertension or hypertension-inducing drugs was a significant risk factor for encephalitis among 90 hospitalized patients in Houston (OR = 2.93; 95% CI 0.97–8.89) (*6*). Additionally, both diabetes and hypertension were predictors of severe illness in 656 WNV patients reported in Colorado in 2003 (*7*). Most recently, in a review of 221 persons hospitalized with WNV infection, West Nile encephalitis was 4 times more likely to develop in patients with diabetes (*8*).

Different mechanisms have been proposed to explain how diabetes and hypertension might promote the development of WNV neuroinvasive disease. Diabetes and its role in impairing immune status may lead to an increase in the magnitude and duration of WNV viremia, while hypertension may cause disruption of the blood-brain barrier, thereby promoting viral entry into the central nervous system (*9*).

This study had several limitations. Local health departments collected case history forms for all reported cases, but only 58% were complete. Case history forms for the severely ill neuroinvasive patients may have been more thoroughly completed than those for West Nile fever patients. Because this surveillance was not designed to specifically evaluate the contribution of diabetes to WNV disease, the information collected on the case history form was limited. However, given the similar findings of other studies, the conclusions from the data are convincing.

In summary, underlying diabetes, as well as older age and male sex, appears to be a significant risk factor for development of WNV neuroinvasive disease. Continued investigation of the role of diabetes, including the degree of severity of diabetic disease, as measured by markers such as degree of end-organ damage (e.g., insulin requirements, presence of diabetic retinopathy, or end stage renal disease), is needed to better identify high-risk patients and target prevention messages.

## References

[1] Glaser CA, Gilliam S, Schnurr D, Forghani B, Honarmand S, Khetsuriani N, In search of encephalitis etiologies: diagnostic challenges in the California Encephalitis Project, 1998–2000. Clin Infect Dis. 2006;36:731–42. 10.1086/367841 12627357

[2] Francisco AM, Glaser CA, Frykman E, Cole B, Cheung M, Meyers H, 2004 California pediatric West Nile virus case series. Pediatr Infect Dis J. 2006;25:81–4. 10.1097/01.inf.0000195612.04911.b2 16395112

[3] O’Leary DR, Marfin AA, Montgomery SP, Kipp AM, Lehman JA, Biggerstaff BJ, The epidemic of West Nile virus in the United States, 2002. Vector Borne Zoonotic Dis. 2004;4:61–70. 10.1089/153036604773083004 15018774

[4] Lethbridge-Çejku M, Vickerie J. Summary health statistics for U.S. adults: National Health Interview Survey, 2003. Vital Health Statistics. Series 10, no. 225 [cited 2007 Oct 23]. Available from http://www.cdc.gov/nchs/products/pubs/pubd/series/ser.htm#sr10 25116227

[5] Nash D, Mostashari F, Fine A, Miller J, O’Leary D, Murray K, The outbreak of West Nile virus infection in the New York City area in 1999. N Engl J Med. 2001;344:1807–14. 10.1056/NEJM200106143442401 11407341

[6] Lillibridge KM, Baraniuk S, Arafat R, Kilborn C, Shallenberger R, Martinez D, Host risk factors for developing encephalitis from West Nile virus infection. International Conference on Emerging Infectious Diseases. Mar 1, 2004; Atlanta, GA, USA. Washington: American Society for Microbiology; 2004.

[7] Patnaik JL, Harmon H, Vogt RL. Follow-up of 2003 human West Nile virus infections, Denver, Colorado. Emerg Infect Dis. 2006;12:1129–31. 16836833 10.3201/eid1207.051399 PMC3291048

[8] Bode AV, Sejvar JJ, Pape WJ, Campbell GL, Marfin AA. West Nile virus disease: a descriptive study of 228 patients hospitalized in a 4-county region of Colorado in 2003. Clin Infect Dis. 2006;42:1234–40. 10.1086/503038 16586381

[9] Campbell GL, Marfin AA, Lanciotti RS, Gubler DJ. West Nile virus. Lancet. 2002;2:519–29. 10.1016/S1473-3099(02)00368-7 12206968

